# Mitochondrial dysfunction in down syndrome: molecular mechanisms and therapeutic targets

**DOI:** 10.1186/s10020-018-0004-y

**Published:** 2018-03-15

**Authors:** Antonella Izzo, Nunzia Mollo, Maria Nitti, Simona Paladino, Gaetano Calì, Rita Genesio, Ferdinando Bonfiglio, Rita Cicatiello, Maria Barbato, Viviana Sarnataro, Anna Conti, Lucio Nitsch

**Affiliations:** 1 Department of Molecular Medicine and Medical Biotechnology, University of Naples Federico II, Via Pansini 5, 80131 Naples, Italy; 2 Institute of Experimental Endocrinology and Oncology, National Research Council, 80131 Naples, Italy; 3 Department of Biosciences and Nutrition, Karolinska Institutet, 17177 Stockholm, Sweden

**Keywords:** Down syndrome/trisomy of chromosome 21, Mitochondrial dysfunction, Mitochondrial dynamics, Chromosome 21 genes, Down syndrome therapy

## Abstract

Trisomy of chromosome 21 (TS21) is the most common autosomal aneuploidy compatible with postnatal survival with a prevalence of 1 in 700 newborns. Its phenotype is highly complex with constant features, such as mental retardation, dysmorphic traits and hypotonia, and variable features including heart defects, susceptibility to Alzheimer’s disease (AD), type 2 diabetes, obesity and immune disorders. Overexpression of genes on chromosome-21 (Hsa21) is responsible for the pathogenesis of Down syndrome (DS) phenotypic features either in a direct or in an indirect manner since many Hsa21 genes can affect the expression of other genes mapping to different chromosomes. Many of these genes are involved in mitochondrial function and energy conversion, and play a central role in the mitochondrial dysfunction and chronic oxidative stress, consistently observed in DS subjects.

Recent studies highlight the deep interconnections between mitochondrial dysfunction and DS phenotype. In this short review we first provide a basic overview of mitochondrial phenotype in DS cells and tissues. We then discuss how specific Hsa21 genes may be involved in determining the disruption of mitochondrial DS phenotype and biogenesis. Finally we briefly focus on drugs that affect mitochondrial function and mitochondrial network suggesting possible therapeutic approaches to improve and/or prevent some aspects of the DS phenotype.

## Background

Down syndrome (DS) is a genetic disorder caused by trisomy of chromosome 21 (TS21) in which the specific phenotypic manifestations may result from the balance among genetic, environmental and stochastic events (Rachidi and Lopes 2007; Reeves et al. 2001). Several studies have demonstrated that TS21 negatively affects mitochondrial function (Valenti et al. 2011; Brooksbank and Balazs 1984; Arbuzova et al. 2002). The downregulation of nuclear encoded mitochondrial genes (NEMGs) has been demonstrated in TS21 fetal heart samples (Conti et al. 2007) and in fetal brains (Mao et al. 2005)*.* The mitochondrial energy production apparatus appears to be less efficient in DS fetal fibroblasts than in controls (Valenti et al. 2011; Valenti et al. 2010; Piccoli et al. 2013). In human primary lines of DS fetal fibroblasts, TS21 demonstrated to perturb the expression of genes involved in mitochondrial pathways, to decrease oxygen consumption and ATP content, to increase mtCa^2+^ load and ROS production (Piccoli et al. 2013; Izzo et al. 2017b). A contemporary dysregulation of ATP translocators (*ANT1*, *ANT2* and *ANT3*) (Piccoli et al. 2013), ATP synthase and adenilate kinase (Valenti et al. 2010) was observed in the same cells*.* Studies of mitochondrial morphology in trisomic fetal fibroblasts demonstrated that these organelles were significantly damaged if compared with those of euploid cells. Furthermore a comparison between tissues from DS fetuses with and without heart defects revealed that TS21 fibroblasts derived from fetuses with cardiopathy presented a more severe mitochondrial dysfunction (Piccoli et al. 2013), thus suggesting that mitochondrial dysfunction contributes to generating a more severe phenotype. This concept might be extended to other phenotypic traits.

Many other studies performed on different DS models, tissues and animals agree with such a mitochondrial scenario in DS. Protein levels of mitochondrial complexes I, III and V were decreased in the brain of DS subjects (Kim et al. 2001) and, moreover, several mitochondrial DNA mutations were found in DS brain tissue (Coskun and Busciglio 2012)*.* Decreased mitochondrial redox activity and membrane potential have been observed in human DS astrocytes and neuronal cultures (Arbuzova et al. 2002; Busciglio et al. 2002; Helguera et al. 2013) as well as in the brain of the Ts1Cje mouse model (Shukkur et al. 2006)*.*

**Fig. 1:**
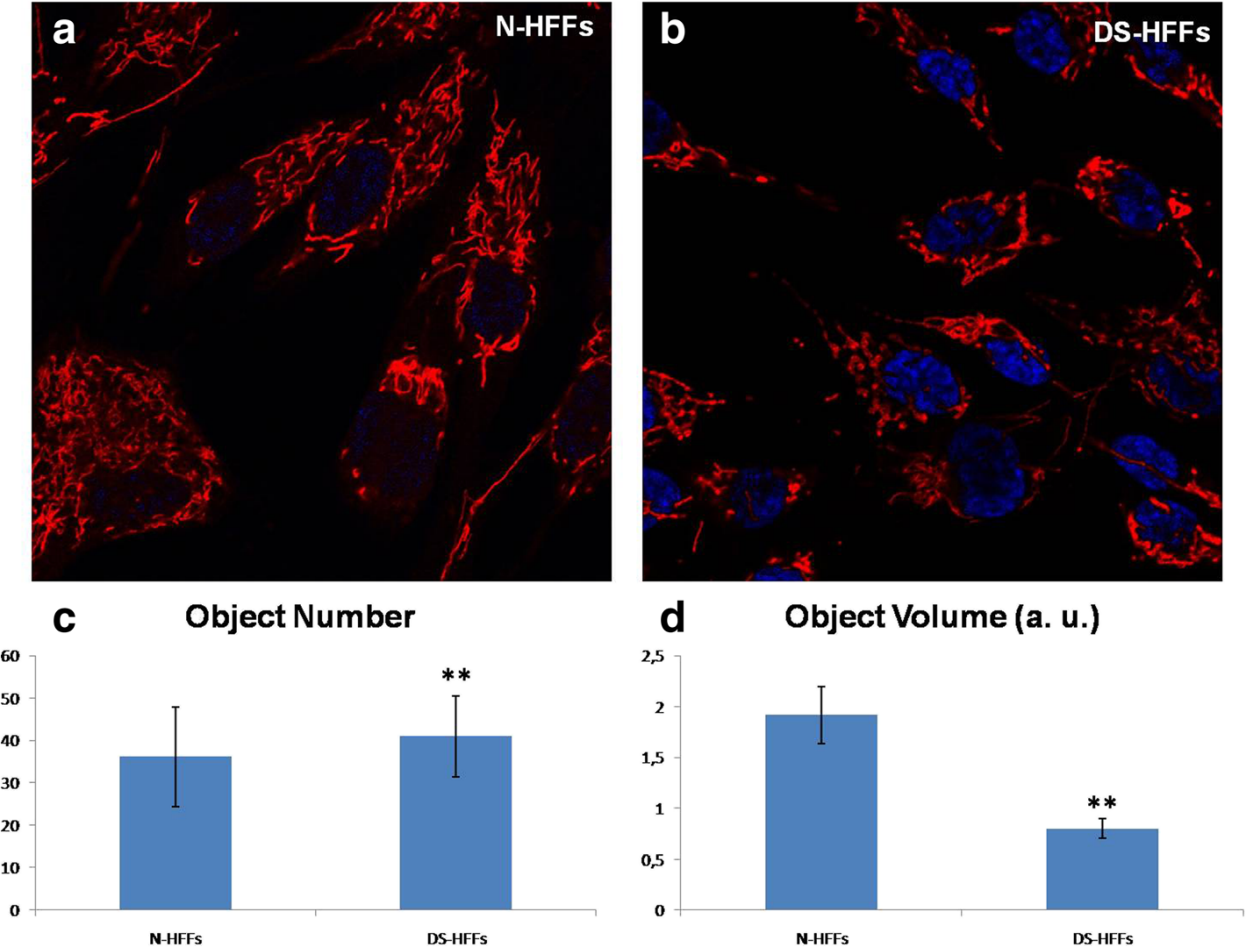
Confocal microscopy of the MitoTracker Red-related fluorescence in N-HFFs versus DS-HFFs. Representative images show that the mitochondrial network is less fragmented in (**a**) N-HFFs than in (**b**) DS-HFFs. **c** The number of mitochondria, measured using the Fiji software (http://www.fiji.sc), is significantly higher in trisomic cells compared with non-trisomic cells. (D) The average mitochondrial volume is significantly lower in DS cells compared with non-trisomic cells. The bars show mean values ± SEM of two non-trisomic and two trisomic cell cultures. Fifteen randomly selected cells for each sample/experimental condition were analyzed. **p ≤ 0.05 Cells were obtained from the ‘Telethon Bank of Foetal Biological Samples’ at the University of Naples. All experimental protocols were approved by the local Institutional Ethics Committee. Abbreviations: N-HFFs: Euploid human fetal fibroblasts; DS-HFFs: Trisomic human fetal fibroblasts; a.u.: arbitrary units.

A substantial alteration in mitochondrial morphology was observed in primary cultures of TS21 human fetal fibroblasts. Mostly, they exhibited reduced or damaged cristae, which were broken, shorter, concentric or highly swollen (Piccoli et al. 2013; Izzo et al. 2017b). Furthermore the mitochondrial network in DS human fibroblasts from Down syndrome fetuses (DS-HFFs) was highly fragmented when compared with euploid cells (N-HFFs) (Fig. 1) with an increased number of shorter mitochondria and a smaller average mitochondrial volume (Izzo et al. 2017b). An increased fragmentation of mitochondrial network was observed also in primary cultures of TS21 astrocytes and neurons (Helguera et al. 2013)*.* It is known that the perturbation of mitochondrial dynamics, as well as of ultrastructure and volume, are mechanistically linked to mitochondrial function (Izzo et al. 2017b).

The observed alterations of the mitochondrial network correlate with the decreased expression of two genes involved in the mitochondrial fusion process, namely *OPA1* and *MFN2* (Izzo et al. 2017b)*.* A decrease in *OPA1* expression is also consistent with the alterations of the proper structure of cristae as *OPA1*, in addition to its role in mitochondrial fusion, is involved in the maintenance and remodeling of cristae morphology (Frezza et al. 2006; Zick et al. 2009).

The common denominator of most of the altered mitochondrial mechanisms is the peroxisome proliferator activated receptor gamma, coactivator 1 alpha (*PGC-1α* or *PPARGC1A*) - a key modulator of mitochondrial function (Scarpulla et al. 2012) – which is significantly downregulated in DS samples (Piccoli et al. 2013) (Conti et al. 2007). Its role in regulating mitochondrial biogenesis and respiratory function is mediated through interaction with transcriptional partners, such as *NRF1*, *ERRa*, *PPARs* and *YY1* that modulate the expression of most NEMGs (Scarpulla et al. 2012). Also these *PGC-1α* transcriptional partners as well as most NEMGs have been found downregulated in DS fetal heart tissue (Conti et al. 2007) and fibroblasts (Piccoli et al. 2013).

In this review, we describe a possible link between the overexpression of Hsa21 genes and mitochondrial dysfunction in DS. We also briefly speculate about possible strategies to restore mitochondrial function and therefore to exert protective effects against DS-associated pathologies.

### Hsa21 genes candidate for inducing mitochondrial alterations in DS

A comprehensive meta-analysis from 45 DS gene expression studies (Vilardell et al. 2011) identified 77 Hsa21 genes mostly upregulated across all the studies. These genes are the ones most likely involved in the DS phenotype. Seven of the genes included in this list, namely *NRIP1*, *SUMO3*, *DYRK1A*, *RCAN1*, *SOD1, APP* and *CBS* are directly or indirectly involved in mitochondrial function (Table 1); therefore their dysregulation might account for mitochondrial alterations observed in DS, as discussed below.

**Table 1:** List of genes mapping to Hsa21 functionally associated to the mitochondrial abnormalities in DS

Hsa21 Genes involved in Mitochondrial function	Effects on mitochondrial phenotype
NRIP1/RIP140	Decreases respiratory efficiency and alters morphology of mitochondria.
SUMO3	Modulates *NRIP1* repressive activity and attenuates the transcriptional activity of *PGC-1α*.
DYRK1A	Controls *PGC-1α* via the *calcineurin/NFAT* pathway.
DSCR1/RCAN1	Controls *PGC-1α* via the *calcineurin/NFAT* pathway and is associated with calcium overloading.
SOD1	Is associated with oxidative stress.
APP	Induces mitochondrial oxidative stress and mitochondrial dysfunction.
CBS	Influences the mitochondrial redox activity negatively regulating methylation processes.
ETS2	Promotes the activation of a mitochondrial death pathway.
ITSN1	Regulates the mitochondrial apoptotic pathway.
PREP1	Inhibits the OXPHOS negatively regulating *PGC-1α* and mitochondrial fusion genes *OPA1* and *MFN2.*
hsa-mir-155	Affects mitochondrial biogenesis by targeting *TFAM*.
hsa-let-7c	May affect mitochondrial function by targeting *ANT1*.

*OXPHOS* Oxidative phosphorylation

We have recently demonstrated that the over-expression of a transcriptional corepressor gene mapping to Hsa21, the nuclear receptor interacting protein 1 (*NRIP1/RIP140*), is responsible for decreased respiratory efficiency and altered morphology of mitochondria in DS (Izzo et al. 2014)*.* Many literature reports corroborate the results of this study (Powelka et al. 2006; Fritah et al. 2010). *NRIP1* was found overexpressed in the hearts and fibroblasts from DS fetuses as well as in other DS tissues (Conti et al. 2007; Piccoli et al. 2013; Vilardell et al. 2011) and *NRIP1* protein was found upregulated in the DS hippocampus (Gardiner 2006). *NRIP1* is known to bind and repress several members of the nuclear receptor superfamily, thus regulating gene expression of NEMGs in adipose tissue, heart, muscle and liver (Fritah et al. 2010; Nautiyal et al. 2013). By these actions the gene significantly affects oxidative metabolism and mitochondrial biogenesis (Powelka et al. 2006). *NRIP1* activity on mitochondrial pathways is mainly exerted through the repressive control on the transcriptional coactivator *PGC-1α* and its targets (Scarpulla 2011) including the transcription factors *NRF1*, *ERRα*, *PPARs*, which are repressed by *NRIP1* and induced by *PGC-1α* in a dose dependent manner (Chen et al. 2012). The PPAR family of nuclear receptor genes consists of three isoforms, *PPAR-α, PPAR-β/δ,* and *PPAR-γ* expressed in different tissues, which induce gene expression through specific interaction with transcription factors. *PPAR-α* and *PPAR-δ* are primarily regulators of lipid oxidation, whereas *PPAR-γ* promotes lipid synthesis and storage. *PPAR-α* cooperates with *PGC-1α* in transcriptional control of nuclear genes encoding mitochondrial fatty acid oxidation enzymes (Vega et al. 2000). In addition to their effects on lipid metabolism, *PPAR-γ* and *PPAR-δ* promote mitochondrial biogenesis in a cell type-specific manner (Hock and Kralli 2009). Till date several PPAR agonists have been tested for their neuroprotective effects against mitochondrial abnormalities in neurodegenerative diseases (Lee et al. 2009; Agarwal et al. 2017; Skerrett et al. 2015). Notably, in all these studies *PPAR-γ* agonists induced the expression of endogenous *PGC-1α*, suggesting that *PPAR-γ* affects mitochondrial biogenesis indirectly by enhancing the transcription of *PGC-1α*. Because *PGC-1α* coactivates *PPAR-γ*, this element also enables *PGC-1α* to enhance its own expression in an autoregulatory fashion (Hock and Kralli 2009).

More than one hundred NEMGs have been found upregulated after *PGC-1α* induction in human osteoblast-like cells (Schreiber et al. 2004). At least 1/3 of these genes are *NRIP1* targets. By contrast, *PGC-1α* null mice (Leone et al. 2005; Mitra et al. 2012), as well as knock-in *NRIP1* mice (Seth et al. 2007), show decreased expression of the same mitochondrial genes in multiple tissues. It appears therefore that *NRIP1* and *PGC-1α* have mutually antagonizing roles in the regulation of metabolism in the tissues where they are co-expressed. Accordingly, we have observed that *NRIP1* is significantly upregulated whereas *PGC-1α* is significantly downregulated in DS fetal hearts and fibroblasts at both mRNA and protein level (Conti et al. 2007; Piccoli et al. 2013)*.* Furthermore, after *NRIP1* silencing the expression levels of *PGC-1α* and NEMGs and consequently the mitochondrial function are restored (Izzo et al. 2014)*.*

Another Hsa21 gene, *SUMO3* affects mitochondrial function by modulating the *NRIP1* repressive activity, as two conserved lysines, Lys756 and Lys1154, located in distinct repression domains of NRIP1, are subject to reversible SUMOylation. The SUMO acceptor lysines have a significant effect on the nuclear distribution of NRIP1 contributing to its corepressor activity (Rytinki and Palvimo 2008). SUMOylation also attenuates the transcriptional activity of PGC-1α, by promoting its transfer outside the nucleus (Rytinki and Palvimo 2009). Even though SUMOylating effects on NRIP1 and PGC-1α have been demonstrated for SUMO-1 and SUMO-2, we presume an identical effect by SUMO-3 that share with SUMO-2 a nearly identical structure (Huq and Wei 2005; Mukhopadhyay and Riezman 2007). SUMO-3 overexpression in DS might therefore be responsible for a concurrent improvement of NRIP1 function and decrease of PGC-1α activity.

The Hsa21 genes, *DYRK1A* and *DSCR1/RCAN1*, have been proposed as possible candidates for mitochondrial abnormalities as they control *PGC-1α* via the calcineurin/*NFAT* pathway (Arron et al. 2006). It is possible that NFAT proteins may exert a positive effect by interacting functionally with MEF2C or MEF2D on the *PGC-1α* promoter (Handschin et al. 2003). Accordingly, in DS fetal hearts and fibroblasts, we have observed that whereas *NFATc3* and *NFATc4* were downregulated, *DYRK1A* and *RCAN1* were upregulated if compared with euploid samples (Piccoli et al. 2013).

*DYRK1A* has also been proposed as relevant candidate gene in various DS phenotypic traits being involved in multiple cellular pathways including neuronal differentiation, nuclear factor activation and basic cellular metabolism (Park et al. 2009).

*RCAN1,* also known as calcipressin, has been found chronically overexpressed in the brain of both DS patients and sporadic AD patients (Sun et al. 2011; Wu and Song 2013)*.* Interestingly, *RCAN1* overexpression has been linked to oxidative stress and mitochondrial dysfunction (Chang and Min 2005; Ermak et al. 2012) and is strictly related to calcium overloading (Sun et al. 2014)*,* as it affects mitochondrial permeability transition pore (mPTP) function. *RCAN1*-induced mPTP opening leads to a series of consequences, including Ca^2+^ retention incapability, massive swelling of mitochondria and rupture of the outer membrane (Sun et al. 2014). In agreement with these data, deregulation of Ca^2+^ homeostasis and Ca^2+^ mediated signaling has been described in cells derived from trisomic patients or in murine models of DS (Caviedes et al. 2006; Yamato et al. 2009). Mitochondrial Ca^2+^ concentration was significantly higher in fetal fibroblasts from DS fetuses than in euploid fetal fibroblasts (Piccoli et al. 2013). Trisomic fibroblasts showed also swelled mitochondria with damaged membranes (Izzo et al. 2017b)*.*

The overexpression of the brain-specific *RCAN1.1S* isoform in mice promotes early age dependent memory and synaptic plasticity deficits, tau pathology, and dysregulation of dynamin-related protein 1 (*DRP1*) activity associated with altered mitochondrial dynamics and oxidative stress (Wong et al. 2015). Duan et al. found that *RCAN1* induces mitochondrial autophagy and improves cell survival in cardiomyocytes (Duan et al. 2015).

A number of studies have provided evidence that the DS phenotype is associated with oxidative stress. The generation of oxidative stress in DS may be attributed to over-expression of some Hsa21 genes even though molecular signals, such as citrate pathway, may play an important role as key metabolic regulators affecting lipid metabolism and ROS production in DS, with a mechanism not yet completely understood (Convertini et al. 2016).

The redox imbalance in DS has been long attributed to overexpression of Cu, Zn superoxide dismutase *SOD1*, which has been investigated in many in vitro, ex vivo and animal studies (Brooksbank and Balazs 1984; Epstein et al. 1987). Levels of *SOD1* in cells from DS patients are approximately 50% greater than normal (Groner et al. 1994). *SOD1*, the major *SOD* in mammalian cells, catalyses the dismutation of superoxide radicals to H_2_O_2_ and O_2_ and is an important antioxidant defence system (Fridovich 1995).

Another gene mapping to chromosome 21 and consistently overexpressed in DS, the amyloid β precursor protein APP, has been proposed to contribute to oxidative stress. Overexpression of *APP* induces mitochondrial oxidative stress and activates the intrinsic apoptotic cascade (Bartley et al. 2012). In addition, amyloid-β fragments, particularly Aβ42, exert direct toxic effects within cells, including Ca^2+^ dysregulation, mitochondrial dysfunction, and induction of oxidative stress (Chen and Yan 2007; Chen and Yan 2010; Demuro et al. 2010). Recent studies have demonstrated that APP protein progressively accumulates within mitochondrial matrix leading to increased free radicals and impaired mitochondrial metabolism (Manczak et al. 2010). In addition *APP* have been shown to translocate to the mitochondria when overexpressed in a human cortical neuronal cell line (Anandatheerthavarada et al. 2003).

The overexpression of genes coding for specific enzymes translates directly into biochemical aberrations that affect multiple metabolic pathways including mitochondrial pathways. An example is given by the alterations that overexpression of the enzyme cystathionine β-synthase (CBS) induces on the methionine/homocysteine pathway in children with DS (Pogribna et al. 2001; Infantino et al. 2011). CBS overexpression indirectly deprives the methionine synthase reaction of one of its precursors, the homocysteine. The significant decrease in plasma methionine levels observed in children with DS may affect the synthesis of S-adenosylmethionine (SAM), the primary methyl donor for cellular methylation reactions. This event is destined to impact mitochondrial redox activity since methylation is a necessary event in mitochondria and relies on the availability and uptake of the methyl donor SAM (Infantino et al. 2011).

Three more Hsa21 genes not included in the list from a meta-analysis by Vilardell (Vilardell et al. 2011) were found to be involved in apoptotic events, namely *ETS-2, ITSN1* and *PKNOX1/PREP1*.

Studies in *ETS-2* transgenic mice show that *ETS-2* overexpression induces apoptosis of thymus, spleen, and brain cells (Wolvetang et al. 2003). Furthermore *ETS-2* promotes the activation of a mitochondrial death pathway in DS neurons (Helguera et al. 2005). Overexpression of *ETS-2* induces cytochrome c cytoplasmic translocation and apoptotic features in normal human cortical neurons (Helguera et al. 2005).

The Intersectin-1 s *(ITSN1)* gene regulates the mitochondrial apoptotic pathway in endothelial cells (Predescu et al. 2007).

Finally, *PKNOX1/PREP1,* another Hsa21 gene encoding for a homeodomain transcription factor, regulates multiple aspects of embryonic development regulating the homeobox protein Pbx activity (Ferretti et al. 2006*).* DS human fibroblasts, which express more *PREP1*, are more sensitive to genotoxic stress. These data suggest that *PREP1* may be involved in the apoptotic phenotype of DS tissues (Micali et al. 2009). Recently it has been demonstrated that *PREP1* regulates mitochondrial oxidative phosphorylation components via direct and indirect mechanisms (Kanzleiter et al. 2014).

Indirectly *PREP1* controls the stability of p160 Myb-Binding Protein, a powerful negative regulator of *PGC-1α* activity (Fan et al. 2004)*.* In the muscle of *Prep1* ablated mice, *Pgc-1α* expression was increased with consequent increase in mitochondrial capacity (Kanzleiter et al. 2014)*.* Furthermore, it has been shown that *PREP1* ablation in muscle leads to an increase in abundance of mitochondrial OXPHOS proteins and an increased citrate synthase activity together with an improved maximal oxidative capacity. ChiP-seq identified *Prep1* binding sites in the promoter regions of genes encoding 16 mitochondrial proteins that were also upregulated in skeletal muscle in response to *Prep1* ablation. This suggests that *PREP1* is a direct negative trascriptional regulator of mitochondrial proteins in addition to its indirect effects via p160-PGC-1α (Kanzleiter et al. 2014)*.* Furthermore, *Opa1* and *Mfn2*, two genes involved in mitochondrial fusion process, were significantly increased in the *Prep1* ablated mice (Kanzleiter et al. 2014)*.* According to these results, *PREP1* negatively regulates mitochondrial proteins thus affecting both mitochondrial function and dynamics.

### Hsa21 miRNAs involved in mitochondrial phenotype

Hsa21 encodes several classes of non-coding RNAs, the most enriched being long non-coding RNAs, while miRNAs are the less represented (Letourneau and Antonarakis 2012). Recent bioinformatic annotations of miRNA database have indicated that Hsa21 harbors 14 miRNAs (Xu et al. 2013)*,* two of them possibly involved in mitochondrial function*.*

It was recently reported that the Hsa21 *miR-155-5p* affects mitochondrial biogenesis by targeting the mitochondrial transcription factor A (*TFAM*) a gene that was found downregulated in trisomic hearts (Quinones-Lombrana and Blanco 2015). TFAM is a nuclear encoded protein that controls the transcription and maintenance of mtDNA and therefore mitochondrial biogenesis.

Another Hsa21 miRNA potential candidate for mitochondrial anomalies is let-7c. By bioinformatics analysis it appears to have several targets among genes that were found downregulated in trisomic fetal hearts and involved in mitochondrial function. Among these targets, we have identified and validated *SLC25A4/ANT1,* a gene that plays a major role in mitochondrial function as it codes for the main translocator of ADP/ATP across the mitochondrial membrane (Izzo et al., 2017a).

These results support the hypothesis that both miR-155 and let-7c dysregulation might have a potential impact on mitochondrial phenotype.

## Conclusions and perspectives

It is now becoming evident that counteracting mitochondrial dysfunction in DS is possible by targeting the *NRIP1- PGC-1α* axis.

It is puzzling that, although drugs capable of modulating the activity of *PGC-1α* and/or the downstream PPAR proteins have been available for a long time, few therapeutic approaches have been so far undertaken to correct the overall mitochondrial dysfunction in DS patients. It is known that *PGC-1α* activity is mainly controlled by *PPARs*, AMP-activated kinases (AMPKs) and the NAD-dependent deacetylase SIRT1 (Canto and Auwerx 2009). Direct phosphorylation by AMPK promotes *PGC-1α* dependent induction at the *PGC-1α* promoter level (Jager et al. 2007), while SIRT1 stimulates *PGC-1α* activity through deacetylation, thereby inducing mitochondrial biogenesis (Rodgers et al. 2005). Pharmacological activators for these proteins, such as metformin, via *AMPK* induction, as well as resveratrol, via *SIRT1* induction, have been tested in mouse models for neurodegenerative diseases in which mitochondrial alterations play a central role such as AD, Parkinson’s disease, Huntington’s diseases, (Jager et al. 2007; Dong et al. 2007; Lagouge et al. 2006). Recent studies indicated that metformin treatment exerts multiple positive effects on mitochondrial activity in a human cell model of DS (Izzo et al. 2017b), while Resveratrol and Epigallocatechin-3-gallate (EGCG) reverse the severe impairment of mitochondrial bioenergetics and biogenesis in hippocampal progenitor cells from the DS mouse model Ts65Dn (Valenti et al. 2016)*.*

Other pharmacological activators of *PGC-1α* pathway, including thiazolidinediones, pioglitazones, and bezafibrates, which selectively stimulate *PPARs* (Landreth et al. 2008)*,* should be tested to restore mitochondrial function in DS.

This will open new therapeutic perspectives to improve the neurological phenotypes in DS and to prevent associated pathologies, such as AD, diabetes, and hypertrophic cardiopathy.

## Abbreviations

AD: Alzheimer’s disease
ANT: Adenine nucleotide translocator
DS: Down Syndrome
DS-HFFs: Human fibroblasts from Down syndrome fetuses
Hsa21: Chromosome 21
mPTP: mitochondrial permeability transition pore
mtDNA: mitochondrial DNA
mΔΨ: mitochondrial membrane potential
NEMGs: Nuclear-encoded mitochondrial genes
N-HFFs: Human non trisomic fetal fibroblasts
OCR: Oxygen consumption rate
OXPHOS: Oxidative phosphorylation
ROS: Intracellular reactive oxygen species
TMRM: Mitotropic probe tetramethylrhodamine methyl ester
TS21: Hsa21 trisomy
